# Caries remineralisation and arresting effect in children by professionally applied fluoride treatment – a systematic review

**DOI:** 10.1186/s12903-016-0171-6

**Published:** 2016-02-01

**Authors:** Sherry Shiqian Gao, Shinan Zhang, May Lei Mei, Edward Chin-Man Lo, Chun-Hung Chu

**Affiliations:** Faculty of Dentistry, The University of Hong Kong, Hong Kong, China

**Keywords:** Caries, Children, Remineralisation, Arrest, Review

## Abstract

**Background:**

As a low-cost and easily operated treatment, the use of professionally applied topical fluoride was approved for preventing dental caries and remineralising early enamel caries or white spot lesions. It is also used to arrest dentine caries. The aim of this study is to investigate the clinical efficacy of professional fluoride therapy in remineralising and arresting caries in children.

**Method:**

A systematic search of publications from 1948 to 2014 was conducted using four databases: PubMed, Cochrane Library, ISI Web of Science and Embase. The key words used were (fluoride) AND (remineralisation OR remineralization OR arresting) AND (children caries OR early childhood caries). The title and abstract of initially identified publications were screened. Clinical trials about home-use fluorides, laboratory studies, case reports, reviews, non-English articles and irrelevant studies were excluded. The full texts of the remaining papers were retrieved. Manual screening was conducted on the bibliographies of the remaining papers to identify relevant articles.

**Results:**

A total of 2177 papers were found, and 17 randomised clinical trials were included in this review. Ten studies investigated the remineralising effect on early enamel caries using silicon tetrafluoride, fluoride gel, silver diamine fluoride or sodium fluoride. Seven studies reported an arresting effect on dentine caries using silver diamine fluoride or nano-silver fluoride. Meta-analysis was performed on four papers using 5 % sodium fluoride varnish to remineralise early enamel caries, and the overall percentage of remineralised enamel caries was 63.6 % (95 % CI: 36.0 % - 91.2 %; p < 0.001). Meta-analysis was also performed on five papers using 38 % silver diamine fluoride to arrest dentine caries and the overall proportion of arrested dentine caries was 65.9 % (95 % CI: 41.2 % - 90.7 %; p < 0.001).

**Conclusion:**

Professionally applied 5 % sodium fluoride varnish can remineralise early enamel caries and 38 % silver diamine fluoride is effective in arresting dentine caries.

## Background

Despite the advance in dental care in the past few decades, dental caries is still a global health problem affecting many children. The US Centers for Disease Control and Prevention reports that 28 % of all US toddlers and preschoolers are affected by caries and that nearly half of US children experience caries before entering kindergarten [1]. Dental caries is the most common chronic disease in US children aged 5-17, and the number is five times higher than that of children who suffer from asthma [2]. Children of social disadvantage such as those from poor families and those whose parents have a low level of education are disproportionately affected [3, 4]. As the prevalence of dental caries is significantly higher for children who come from lower social-economic classes or lower income families, conventional dental care is often either unavailable or unaffordable for them [5, 6]. Moreover, the lack of dental manpower and the sophisticated dental equipment required make conventional restorative care a difficult way to solve the caries problem. Alternative treatments for dental caries of children in disadvantaged communities are therefore necessary, and professionally applied fluoride therapy has been proposed for the management of dental caries [7].

Contemporary caries management philosophy has changed from the traditional surgical approach to a medical model, and fluoride therapy is now used not only to prevent but also to arrest caries. Professionally applied fluoride therapy is a relatively low-cost and easily operated treatment and has been used to arrest active dental caries [7]. Fluoride inhibits enamel demineralisation. The calcium fluoride that is deposited onto a tooth surface after fluoride therapy is not readily soluble and can act as a fluoride reservoir [8]. This fluoride also can lower the critical pH value of hydroxyapatite crystal dissolution, or the pH value when demineralisation occurs, from approximately 5.5 to 4.5 in the mouth. Fluoride can be incorporated incrementally into fluorapatite crystals on the tooth surface, making the surface more resistant to acid dissolution. In addition to inhibiting demineralisation, fluoride enhances enamel remineralisation, increasing the speed of the remineralisation process and the mineral content of early carious lesions. The incorporation of fluoride also makes the deposited mineral less acid soluble. Although fluoride’s specific mechanism of action in caries prevention is not fully understood, it is generally accepted that topically applied fluorides have an effect on tooth surfaces. Fluoride inhibits plaque metabolism, alters plaque composition, affects plaque formation and reduces plaque bacteria’s ability to produce a large amount of acid from carbohydrates [8].

Different types and forms of fluoride agents in various concentrations are used in dentistry. Many studies have reported their effectiveness in preventing dental caries among children and adolescents [9]. Furthermore, the use of professionally applied topical fluoride was extended to remineralise early enamel caries or white spot lesions and arrest dentine caries. Although the caries remineralising or arresting effect was demonstrated in literature, until now, there has been no comprehensive and systemic review to evaluate the level of evidence. The aim of this study is to systematically review the clinical efficacy of professionally applied fluoride therapy in remineralising and arresting dental caries for children.

## Methods

The PRISMA statement for reporting systematic review and meta-analysis of studies was used in this review [10].

### Search strategy

A systematic search of the publications was performed by two investigators (SSG and SZ) separately on four databases: Cochrane Library, Embase, ISI Web of Science and PubMed. Search key words are (fluoride) AND (arresting OR remineralisation OR remineralization) AND (children caries OR early childhood caries). Articles from 1948 to 2014 that contained the search terms were selected out to generate a potentially eligible list, which was included in this review for the first screening.

### Selection of clinical studies

Publications in the potentially eligible list were searched manually, and title and abstract were screened. Clinical studies about over-the-counter or home-use fluoride products, reviews, discussion papers, laboratory works, case reports, clinical treatment, non-English articles and irrelevant studies were excluded. Full texts of the remaining publications were retrieved. Manual search was performed on the bibliographies of these publications to identify relevant papers, which were included for assessment. Finally, studies that met the following criteria were selected in this systematic review: the study type is clinical trial on children and the outcome measurement of the studies should be evaluating the remineralisation or arresting effect of caries by professional fluoride treatment. Studies that met the criteria above were included for data analysis.

After finishing screening, the two investigators discussed the selected articles. If there were questions, the article was discussed with the third investigator (CHC) before making a decision. Data were categorised into two groups (remineralising early enamel caries and arresting dentine caries). The percentage of remineralised early enamel caries and the percentage of arrested dentine caries were calculated. The risk of bias of each study was evaluated separately by the two investigators (SSG and SZ), and the results were discussed with the third investigator (CHC).

### Data collection and analysis

Data of the selected studies were divided into two groups, early enamel caries and dentine caries, before statistical analysis. For studies investigating early enamel caries, the total number of caries lesions before and after the treatment was obtained from the original data reported by the researchers. Then, the percentage of remineralised early enamel caries in each study was calculated. Meta-analysis (Stata 13.1, StataCorp LP, Texas, USA) using the random-effects model was used to evaluate the overall percentage of remineralised early enamel caries and to show the effective weight of each study in this review according to the sample size and calculated percentage of remineralised early enamel caries. For studies investigating dentine caries, the total number of active dentine caries surfaces at baseline and the total numbers of arrested dentine caries surfaces after intervention were used to calculate the caries arresting rates. According to method in the included studies, caries was recorded as active when it was soft by gentle probing and arrested when it was hard to probing [11–17]. Meta-analysis using the random-effects model was used to compare the proportion of dentine caries being arrested as the caries arresting proportion. The risk of bias of each study was performed according to the method suggested by the Cochrane Handbook for Systematic Review of Interventions [18].

## Results

**Fig. 1:**
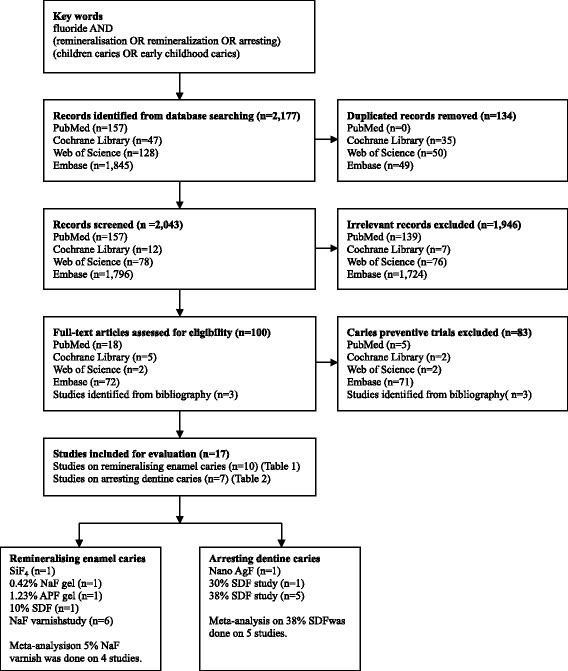
Flow chart of the literature search

The initial search identified a total of 2177 articles from 1948 to 2014 in the four databases. There were 157 articles found from PubMed, 47 articles from Cochrane Library, 128 articles from ISI Web of Science and 1845 articles from Embase (Fig. 1). Among them, 134 papers were duplicate records and were removed. After screening the title and abstract manually, two investigators reached an agreement that 97 publications on clinical trials met the inclusion criteria, with 1946 publications excluded because of being classified as trials on home-use or over-the-counter fluoride products, laboratory study, case report, literature review, etc. The full texts of the 97 publications were retrieved and 3 potentially relevant publications identified from the bibliographies were added. As a result, the full texts of 100 publications were reviewed.

After the full review, 83 articles were removed because those studies investigated caries prevention by measuring the reduction of new caries development over the study period (Fig. 1). The remaining 17 studies were categorised into 2 groups for assessment. Group 1 had ten studies, which investigated the use of professional fluoride application to remineralise early enamel caries or white spot lesions (Table 1). Among them, a study used silicon tetrafluoride and found it could not remineralise white spot lesions [19]. Two studies used fluoride gel. One of them used sodium fluoride gel and the result showed a significant remineralising effect on early enamel caries [20]. The other study used acidulated phosphate fluoride gel and the result could not find the gel effective to remineralise early enamel caries [21]. A study found 10 % silver diamine fluoride therapy, glass ionomer restoration and tooth-brushing had similar effect in remineralising early enamel caries [22]. Six studies investigated sodium fluoride (NaF) varnish and they found it could remineralise early enamel caries [23–28]. The risk of bias of the studies is shown in Table 1.

**Table 1:**
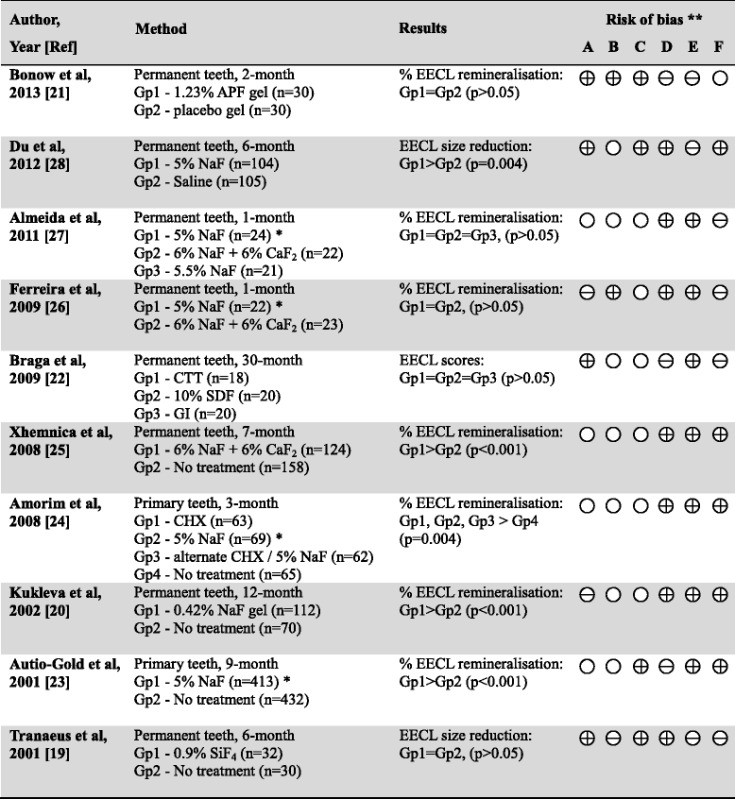
Summary of trials of professionally applied fluorides in remineralising early enamel caries

*EECL* Early enamel caries lesion, *NaF* Sodium fluoride, *CaF*_*2*_ Calcium fluoride, *CHX* chlorhexidine at 1 %, *SiF*_*4*_ Silicon tetrafluoride, *APF* acidulated phosphate fluoride, *GI* glass ionomer, *CTT* cross tooth-brushing technique

**Fig. 2:**
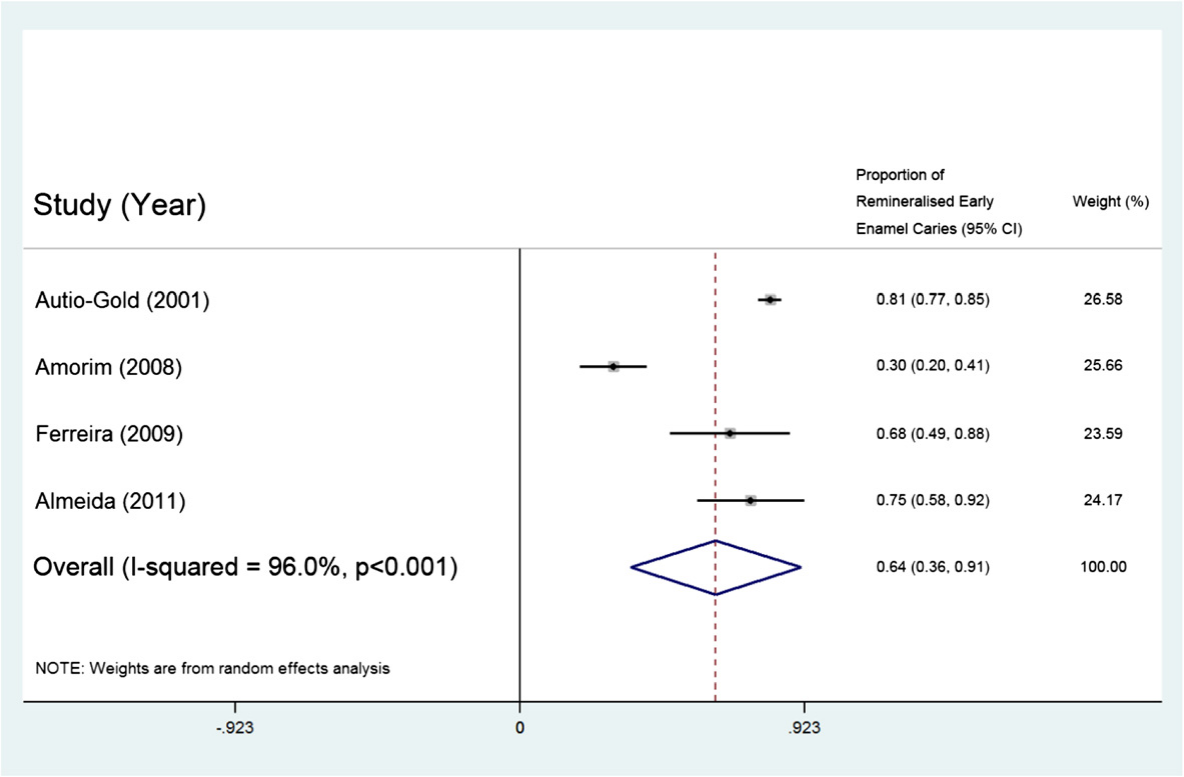
Meta-analysis of the 4 studies using 5 % NaF to remineralise early enamel caries

* Data included for meta-analysis. (Fig. 2)

** Risk of bias legend:

(A) Random sequence generation (selection bias)

(B) Allocation concealment (selection bias)

(C) Blinding of outcome assessment (detection bias)

(D) Incomplete outcome data (attrition bias)

(E) Selective reporting (reporting bias)

(F) Other bias

= Low risk,  = High risk,  = Unclear risk

Only one study of NaF reported the dimension change of caries lesion, but five studies of NaF reported the percentage of remineralised early enamel caries. Meta-analysis was performed on four of these five studies, because one study that tested 6 % NaF with 6 % calcium fluoride was excluded [25]. The results showed that the overall percentage of remineralisation of early enamel caries was 63.6 % (95 % CI: 36.0 % - 91.2 %; p < 0.001) (Fig. 2). Although it could not be included in the meta-analysis, a clinical trial that evaluated dimensional change of the caries lesion [28] showed that the average size of early enamel caries in children receiving professional NaF therapy was significantly smaller than in those with no fluoride treatment. Another study also reported a significant reversal of early enamel caries by using 6 % NaF with 6 % calcium fluoride compared with no treatment [25].

**Fig. 3:**
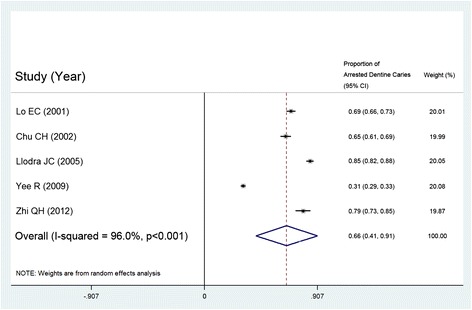
Meta-analysis of the 5 studies using 38 % SDF to arrest dentine caries

Group 2 comprised studies investigating the arresting effect of fluoride in dentine caries (Table 2). There were seven studies and they investigated the use of silver diamine fluoride solution (SDF) or nano-silver fluoride solution in children. When referring to the 7 dentine caries studies, 38 % SDF, 30 % SDF, 12 % SDF and nano-silver fluoride all showed an obvious effect in arresting dentine caries. There were 5 studies using SDF at 38 % in arresting dentine caries, and they were included in meta-analysis. Three studies reported annual application of SDF [11, 12, 14] and their mean proportion of arrested dentine caries ranged from 65.2 % to 79.2 %. One study used 38 % application every 6 months, and the mean proportion of arrested dentine caries was 84.8 % [13]. Another study used 38 % SDF as a one-off application at baseline and the mean proportion of arrested dentine caries was 31.2 % [15]. The overall proportion of arrested dentine caries after SDF solution treatment was 65.9 % (95 % CI: 41.2 % - 90.7 %; p < 0.001) (Fig. 3), with every study presenting a relatively equivalent weight. The risk of bias of the studies is shown in Table 2.

**Table 2:**
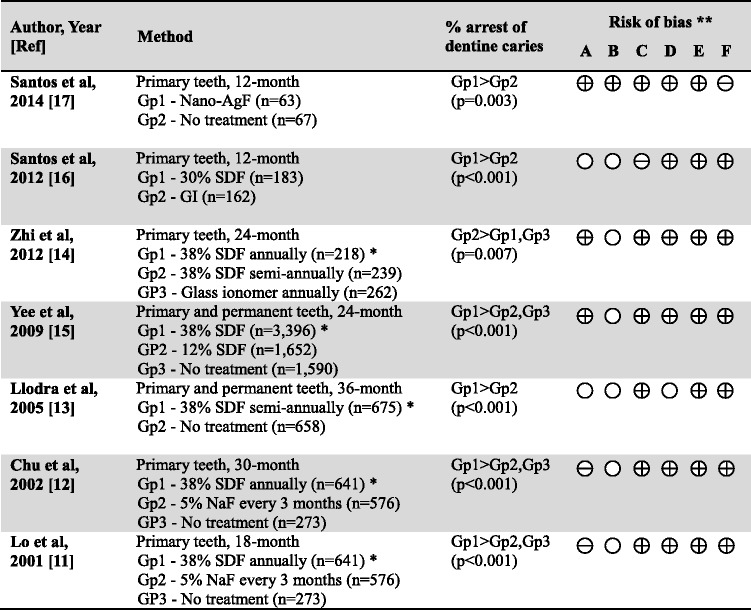
Summary of trials of professionally applied fluorides in arresting dentine caries

*Nano-AgF* Nano silver fluoride, *SDF* Silver diamine fluoride, *GI* Glass ionomer

* Data included for meta-analysis. (Fig. 3)

** Risk of bias legend:

(A) Random sequence generation (selection bias)

(B) Allocation concealment (selection bias)

(C) Blinding of outcome assessment (detection bias)

(D) Incomplete outcome data (attrition bias)

(E) Selective reporting (reporting bias)

(F) Other bias

= Low risk,  = High risk,  = Unclear risk

## Discussion

Two Cochrane reviews on caries prevention of professionally applied fluoride (gels and varnishes) were published and they showed that professionally applied fluoride is effective in caries prevention [29, 30]. Apart from preventing dental caries, professionally applied fluoride is also used by clinicians to remineralise early enamel caries and to arrest dentine caries. However, there is no systemic review on professionally applied fluoride to arrest dental caries. It is essential to investigate the clinical efficacy of professional fluoride therapy in remineralising and arresting caries in children. Over the years, researchers established standards such as CONSORT 2010 [31] and SPIRIT 2013 statement [32], with the goal to produce up-to-standard clinical trials. Although this systematic review was performed with four mainstream databases, the number of clinical trials included in this review were not many. In addition, the methodology and outcome measurement varied between studies, making comparison difficult. Hence, not all selected studies were able to be included in meta-analysis. The amount of caries at baseline was different between studies. Therefore, the number (absolute values or delta changes) of the arrested caries was not used for analysis in this review. In this study, the proportion of remineralised early enamel caries and arrested dentine caries were used for meta-analysis. The risk of bias of each study was evaluated with six legends according to the Cochrane Handbook of Systematic Review of Interventions [18]. For some studies, blinding of outcome measurement and allocation concealment were either not achieved or not mentioned by the researchers. The sample size of some studies was small, while some studies didn’t report the statistical procedure of sample size calculation or justified the sample size used in their studies. Moreover, clinical trials with favourable results generally have more opportunity to be published than those with insignificant outcomes. Such a publication bias may lead reviewers to draw a positive conclusion. In this review, two independent reviewers (SSG and SZ) performed screening of the literature independently using well-defined inclusion criteria to minimise the selection bias. Last but not least, only English articles were considered in this review. SDF is mainly used in Asian countries such as Japan and China and in South American countries such as in Brazil and Argentina [33]. This review may not be comprehensive because those studies published in Japanese, Chinese, Spanish or Portuguese were not included.

Meta-analysis would provide more reliable and less biased results because of inclusion of many studies [34]. It provides more reliable estimates by combining the information of a number of independent studies and conducts a statistic analysis of various results. Although meta-analysis is now widely used in gathering information from different clinical trials, it requires trials to have similar outcome measurement and be uniform in result presentation. Some clinical trials cannot be selected because of variations in the outcome measurements. Moreover, it is difficult to standardise all published clinical trials, and the influence of between-trials heterogeneity is uncertain [35]. As the studies included in this review were conducted by different researchers using not exactly the same treatment, the random-effects model was used in the test. By using the random-effects model, studies with larger sample sizes were weighed more in the analysis and thus have a greater influence on the overall result.

The effectiveness of NaF varnish in preventing dental caries was reported in other reviews [36, 37] and is not the aim of this review. This review looked into the studies on remineralisation of early enamel caries or white spot lesions. Six clinical trials were included in this review and they all demonstrated that NaF could remineralise early enamel caries. Results of meta-analysis on four studies showed that 5 % NaF varnish remineralised approximately two-thirds of early enamel caries lesions in children [23, 24, 26, 27]. Additional ingredients or chemical agents such as chlorhexidine or calcium fluoride were added into the NaF varnish, but the addition had no significant effect on remineralising early enamel caries [24, 26, 27]. Apart from NaF varnish, there is limited evidence to support the benefits of using other professional-applied fluoride agents such as 0.9 % silicon tetrafluoride [19], 0.42 % NaF gel [20, 21] and 10 % SDF [22] in remineralising early enamel caries.

Neutral silver fluoride can be unstable [38]. Therefore, it is dissolved in ammonia to form complex ions diammine silver fluoride, and is therefore referred to as SDF by some researchers [38]. The most commonly used concentration of SDF is 38 % (44,800 ppm F), but other concentrations of SDF solutions at 30 % (35,400 ppm) and 12 % (14,150 ppm) were also used to manage caries [39]. Five studies were selected in this systematic review. They all concluded that SDF is more effective in caries prevention than fluoride varnish [40]. It is noteworthy that the meta-analysis was derived from the all-positive findings and the results should be interpreted with care. In this review, 38 % SDF was found effective to arrest dentine caries among children for both primary and permanent teeth. Studies found that 38 % SDF treatment is superior to NaF varnish in arresting dentine caries [11, 12]. There is no need to remove the soft decay (the infected dentine) before SDF application [12]. In a clinical trial done in Nepal, a one-off application of SDF was applied at baseline [15], while in other studies, SDF application was generally applied once a year to children. This could be one of the main reasons for a lower caries arrested rate than other studies. Another trial found that increasing the frequency of 38 % SDF treatment from annual application to twice a year would increase the caries arresting rate [14]. Two studies reported that SDF was superior to glass ionomer restorations in arresting dentine caries [14, 16]. But it should be noted that there was no removal of carious tissue before glass ionomer restoration and the restorative procedure was performed in a field where moisture control was compromised. Apart from 38 % SDF, 30 % SDF was also reported as an effective intervention to arrest dentine caries on primary teeth [16]. However, a one-off application of 12 % SDF was found ineffective to arrest caries [15]. A limitation in this review is there are only a few clinical trials reported in the literature. The five publications selected had different duration, dentition, starting age, mode of delivery, concentration and frequency of SDF treatment.

Black staining of caries lesions after SDF application is an important disadvantage, which may cause dissatisfaction of the children and their parents. A study used nano-silver fluoride as an innovative product and found it was effective to arrest dentine caries without causing dark staining [17]. SDF at 38 % contains a high concentration of silver and fluoride. The safety issue of its use in particular in young children can be an important concern. The existing literature, however, reported no serious adverse effect. More studies on this aspect should be performed in the future.

## Conclusion

In conclusion, professionally applied 5 % sodium fluoride varnish shows the capability to remineralise early enamel caries in children. Silver diamine fluoride solution at 38 % is effective in arresting active dentine caries. Because the number of clinical trials that studied the arresting effect of dental caries is limited, more clinical trials should be performed.

## Abbreviations

NaF: Sodium fluoride
SDF: Silver diamine fluoride
